# Efficient Biofilm-Based Fermentation Strategies for L-Threonine Production by *Escherichia coli*

**DOI:** 10.3389/fmicb.2019.01773

**Published:** 2019-08-02

**Authors:** Tianpeng Chen, Na Liu, Peifang Ren, Xun Xi, Leyun Yang, Wenjun Sun, Bin Yu, Hanjie Ying, Pingkai Ouyang, Dong Liu, Yong Chen

**Affiliations:** ^1^State Key Laboratory of Materials-Oriented Chemical Engineering, College of Biotechnology and Pharmaceutical Engineering, Nanjing Tech University, Nanjing, China; ^2^Jiangsu National Synergetic Innovation Center for Advanced Materials, Nanjing Tech University, Nanjing, China

**Keywords:** *Escherichia coli*, L-threonine, *fimH* gene, biofilm, transcriptome analysis

## Abstract

Biofilms provide cells favorable growth conditions, which have been exploited in industrial biotechnological processes. However, industrial application of the biofilm has not yet been reported in *Escherichia coli*, one of the most important platform strains, though the biofilm has been extensively studied for pathogenic reasons. Here, we engineered *E. coli* by overexpressing the *fimH* gene, which successfully enhanced its biofilm formation under industrial aerobic cultivation conditions. Subsequently, a biofilm-based immobilized fermentation strategy was developed. L-threonine production was increased from 10.5 to 14.1 g/L during batch fermentations and further to 17.5 g/L during continuous (repeated-batch) fermentations with enhanced productivities. Molecular basis for the enhanced biofilm formation and L-threonine biosynthesis was also studied by transcriptome analysis. This study goes beyond the conventional research focusing on pathogenic aspects of *E. coli* biofilm and represents a successful application case of engineered *E. coli* biofilm to industrial processes.

## Introduction

L-threonine is one of the most essential amino acids in human body, and its demand is sharply increasing due to its wide application in food, chemical, and pharmaceutical industries (Leuchtenberger et al., 2005). Currently, microbial fermentation is widely employed for industrial L-threonine production with *Escherichia coli* as the best candidate strain (Lee et al., 2006; Dong et al., 2011). However, L-threonine fermentation has been operated in a free-cell batch fermentation mode, wherein cells cannot be reused after fermentation (Rajkumar et al., 2013; Boelee et al., 2014). This batch fermentation and single-use of cells would increase the cost of operation and reduce productivities. Meanwhile, the free cells dispersed in fermentation media are often challenged by stress conditions such as shear forces during aerobic fermentation, resulting in decreased cell viability over the fermentation process. These problems need to be solved urgently to improve the fermentation efficiency. Alternatively, biofilm-based immobilized fermentation has been proposed as an alternative to free-cell fermentation owing to its advantages such as protection by biofilm matrix, enhanced metabolic activities, and repeated use of cells compared with free-cell fermentation processes (Zhao et al., 2015). The biofilms of some microorganisms such as *Clostridium acetobutylicum*, *Corynebacterium glutamicum*, *Aspergillus niger*, and *Saccharomyces cerevisiae* have been applied to immobilized batch or continuous (repeated-batch) fermentation effectively (Liu et al., 2013; Shi et al., 2014; Yang et al., 2018; Yu et al., 2018). However, for *E. coli*, one of the most important platform strains, industrial application of the biofilm has not yet been reported, though the biofilm has been extensively studied for pathogenic reasons.

Biofilms are complex cell communities living in close association with biological or abiotic surfaces (Sauer, 2003). For pathogenic bacteria, formation of biofilms is one of the most important factors leading to medical infection which is difficult to be removed (Stoodley et al., 2002). Type I fimbriae is one of the most important factors for biofilm formation in Gram-negative bacteria such as *E. coli* (Tripathi et al., 2013). In *E. coli*, a *fimH*-encoded protein that is secreted and located at the top of type I fimbriae plays a key function to generate biofilm structures by serving as an adhesin (Nishiyama et al., 2008; Le Trong et al., 2010). Cells could use these structures to obtain nutrients and withstand shear forces. It was found that *E. coli* cells covered by biofilms could tolerate stricter conditions such as high osmotic pressure, oxygen limitation, and high cell density, which is a desired characteristic during fermentation (Prigent-Combaret et al., 1999; Weissman et al., 2006).

**FIGURE 1:**
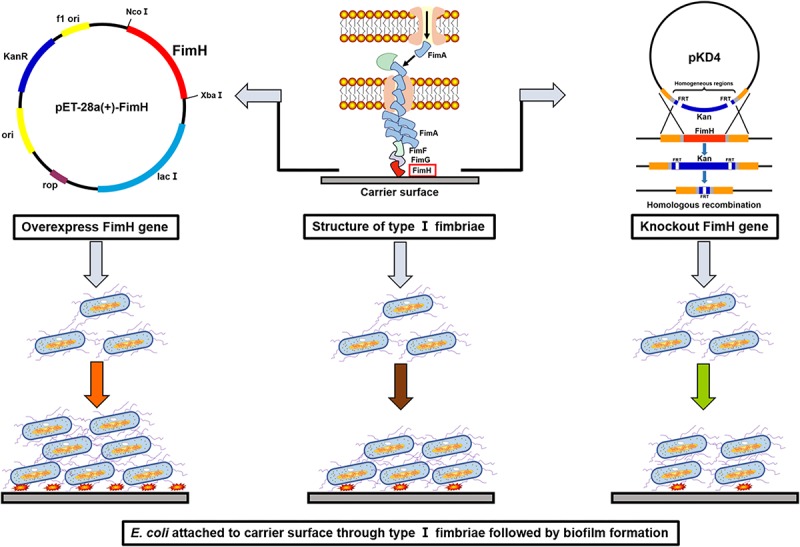
A schematic illustration of constructions for the overexpression or knockout of *fimH* in *Escherichia coli* W1688 and the effects on the biofilm formation.

In this study, *E. coli* was first metabolically engineered with overexpression of *fimH* gene to enhance biofilm formation (Figure 1). A biofilm-based fermentation system was constructed using a carrier to support the biofilm. Cells adhered to the surface of the carrier and formed a large amount of biofilm so that it could withstand high-speed shaking. Moreover, the biofilm cells that attached to the carrier surface could be renewed when the fermentation broth was replaced with fresh medium (Huang et al., 2002; Kim et al., 2014). Due to high cell activities and repeated use of cells in the biofilm-immobilization fermentation, no seed culture was needed and cellular lag phase and fermentation period were reduced substantially. Overall, this study represents a successful case of development of biofilm-based immobilized fermentation under aerobic industrial conditions for efficient biochemical production.

## Materials and Methods

### Strains and Plasmids

*Escherichia coli* W1688 (CCTCC M2015233) was an L-threonine producer obtained from *E. coli* MG1655 (ATCC47076) by mutation and molecular modification. It could not produce biofilms apparently. All strains and plasmids used in this work are listed in Tables 1, 2, respectively. The *fimH* gene was amplified from the genomic DNA of *E. coli* W1688. The *fimH* gene and plasmid pET28a (with restriction enzyme *Xba*I and *Nco*I) were ligated by using the ClonExpress II One Step Cloning Kit C112-01 (Vazyme, Nanjing, China), resulting in a plasmid pET28a-*fimH*. The final engineered strain was named *E. coli* W1688-fimH^*^ with Kanamycin resistance for screening. On the other hand, *fimH* from *E. coli* W1688 was deleted by Red homologous recombination, resulting in an *E. coli* W1688-ΔfimH (Madyagol et al., 2011). Briefly, a PCR-generated Kanamycin resistance marker was used as knock-in DNA fragment. The Kanamycin resistance marker consisted of a Kanamycin resistance sequence in plasmid pKD4 and homologous regions (50–100 bp) flanking the target locus. The knock-in component was transformed into strain *E. coli* W1688-pKD46 using Bio-Rad electroporation system set at 2.0 kV, 25 mF with a 200 Ohm pulse controller.

**TABLE 1:** Strains and plasmids used in this study.

**Strains or plasmids**	**Relevant characteristics**	**Sources**
Strains		
*E. coli* W1688^a^	L-threonine producing strain	Prof. Sheng Yang
*E. coli* W1688-fimH^*^	*E. coli* W1688 harboring plasmid pET28a-*fimH*	This study
*E. coli* W1688-ΔfimH	*E. coli* W1688 with the deletion of *fimH*	This study
*E. coli* W1688-pKD46	*E. coli* W1688 harboring plasmid pKD46	This study
Plasmids		
pET28a	Kan resistance	Stored in our lab
pET28a-*fimH*	pET28a containing *fimH*	This study
pKD46	Amp resistance	Stored in our lab
pKD4	Kan resistance	Stored in our lab

*^*a*^A gift from Prof. Sheng Yang (Institute of plant physiology and ecology, CAS, Shanghai, China).*

**TABLE 2:** Primers used in this study.

**Primer**	**Sequence**
fimH-F	ATGAAACGAGTTATTACCCTG
fimH-R	GTGACTTTTGTTTATCAATAA
fimH-Kan-F	GATTAGCATCACCTATACCTACAGCTGAACCCGAAGAGAT GATTGTAATGGTGTAGGCTGGAGCTGCTTC
fimH-Kan-R	TACCAGCATTAGCAATGTCCTGTGATTTCTTTATTGATAAA CAAAAGTCAGCCATGGTCCATATGAATATCCTCC
fimH-Kan-cx-F	GTGTAGGCTGGAGCTGCTTC
fimH-Kan-cx-R	GCCATGGTCCATATGAATATCCTCC
fimH-mq-F	GGATAACAATTCCCCTCTAGAATGAAACGAGTTAT TACCCTGTTTG
fimH-mq-R	GATGATGGCTGCTGCCCATGGTTATTGATAAACAAA AGTCACGCCA
fimH-cx-F	TATAGGCGCCAGCAACCGCACC
fimH-cx-R	CCGCGACCCATTTGCTGTCCA

### Media and Growth Conditions

*Escherichia coli* W1688, *E. coli* W688-fimH^*^ and *E. coli* W1688-ΔfimH were cultured in LB medium, which contained 5 g/L yeast extract, 10 g/L tryptone and 10 g/L NaCl. Solid media was prepared in all cases by adding 1.5% (w/v) agar. Fermentation medium contained 30 g/L glucose, 2 g/L yeast extract, 1 g/L KH_2_PO_4_, 20 g/L (NH_4_)_2_SO_4_, 0.8 g/L MgSO_4_⋅7H_2_O, 0.2 g/L FeSO_4_⋅37H_2_O, 0.2 g/L MnSO_4_⋅5H_2_O, and 15 g/L CaCO_3_. Acetic acid was added to the medium to adjust its initial pH to 7.2. All media were sterilized at 115°C for 20 min. Kanamycin (50 mg/mL), ampicillin (100 mg/mL), isopropyl-β-D-thiogalactopyranoside (IPTG) 0.5 mM or L-arabinose (100 mM) were added as necessary. The fermentation culture was grown at 37°C with a shaking speed of 200–220 rpm.

### Carrier Preparation

A new type of polymer porous foam named Y-11 which was made of polyurethane, was prepared in the laboratory. The carrier Y-11 had a density of 0.32 g/cm^3^ with a pore diameter of 0.2 to 0.6 mm sheared to a size of 10 mm × 10 mm × 10 mm. This carrier was pretreated with the previously reported method. The carriers were rinsed in 1 M NaOH and then1 M HCl before being washed by ddH_2_O (sterile water) until the pH-value reached 7.0. All carriers were sterilized at 115 C for 20 min (Zhao et al., 2015).

### Free-Cell Fermentation and Immobilized Fermentation

For free-cell fermentation, the flasks were inoculated with 5% seed culture and then run at 37°C with shaking at 220 rpm. Samples were centrifugated at 8000 rpm, 4°C for 5 min, then the supernatants were used for the quantification of L-threonine and residual sugar.

For immobilized continuous (repeated-batch) fermentation, the same conditions for free-cell fermentation were employed and 30 g/L of the carrier was added into the fermentation medium. After the first batch, 80% of the fermented broth was removed from the flask and 20% of the broth with the carrier that was covered by biofilm was left for the second batch. After adding fresh culture medium, the second batch was initiated under the same conditions described above until the L-threonine titer was stable. The subsequent batches were operated in the same way as above.

### Analytical Methods

L-threonine concentrations were measured by high-performance liquid chromatography (Agilent 1260 series; Hewlett-Packard, Palo Alto, CA, United States) with a UV detector, using a Sepax AAA ion exclusion column (250 × 4.6 mm; Bio-Rad Laboratories, Hercules, CA, United States), with 0.1 M sodium acetate and 80% acetonitrile as the mobile phase (1 mL/min) at 36°C. Glucose concentrations were measured by a refractive index detector, using an Aminex HPX-87H column (300 × 7.8 mm), with 5 mM H_2_SO_4_ as the mobile phase (1 mL/min) at 55°C.

The basic dye crystal violet binds to negatively charged surface molecules and polysaccharides in the biofilm matrix which can be applied for total biomass staining (Li et al., 2003). Strains were grown in LB medium or fermentation medium at 37°C for 12 h. Then they were diluted to the ratio of 1:200 in 96-well plates (200 μL in each well) and kept at 37°C without shaking. PBS (1%) was prepared for washing the biofilm-containing wells twice to remove free cells. Methanol was used for fixing biofilm for 15 min at 4°C. Biofilms were then stained with 200 μL of 1% crystal violet for 15 min at 37°C, after which wells were washed by PBS 3–4 times repeatedly. For quantification, 200 μL of 33% acetic acid was added to release the crystal violet and the plates were incubated by shaking slowly for 30 min. The absorbance of crystal violet was an indicator of biofilm amount and was measured at 570 nm by using a multiscan spectrum (SpectraMax^®^ iD5; Molecular Devices; United States).

In some cases, coverslips were used as carriers to observe biofilms by microscopy. The coverslips were removed gently from the fermentation broth after culture of 30 h. PBS (1%) was used to wash off the free cells on the coverslips. The biofilm was fixed by 4% paraformaldehyde for 12 h at 4°C. The coverslips with the biofilm were dehydrated by vacuum freeze-drying device (Labconco Corporation, Fort Scott, KS, United States), then coated with gold-palladium before scanning electron microscopy (SEM) analysis (TM3000, Hitachi, Japan). Fresh carriers or carriers after the continuous (repeated-batch) fermentation were prepared in the same way as above for SEM analysis.

Fluorescence microscope was used to visualize the adhesion property of bacteria and distribution of cells in biofilms. The carriers were removed gently from the fermentation broth after culture for 30 h. PBS (1%) was used to wash off the free cells. The biofilm was fixed by 4% paraformaldehyde for 30 min at 4°C. Then 0.2 μg/mL of 4′,6-Diamidino-2-phenylindole (DAPI) (Sigma, St. Louis, MO, United States) was prepared to stain the cells in the biofilm for 30 min at room temperature.

### Transcription Analysis

Samples of the three different strains were centrifuged at 8000 rpm for 5 min at 4°C after 30 h of immobilized batch fermentation. Three biological replicates were prepared. Sediments were collected and washed twice with PBS, and then frozen in liquid nitrogen immediately and stored at −80°C. The level of false discovery rate (FDR) ≤ 0.05 and absolute value of Log_2_Ratio ≥ 1 were selected as criteria for assessing expression levels of different genes.

### Quantitative Reverse Transcription PCR (qRT-PCR) Analysis

The *E. coli* wild-type strain and two recombinant strains were harvested at exponential phase. Total RNA was isolated using RNAprep Pure Cell/bacteria Kit (TianGen Biotech, China) and the residual DNA was digested by using TianGen RNase-Free DNase. After reverse transcription, cDNAs were prepared for qRT-PCR. Primer Express software was used for primer design. The analyzed genes and primers used in the analysis are listed in Table 3. qRT-PCR assays were performed by using SYBR Green PCR Master Mix (Applied Biosystems, United States) in a StepOnePlus Real-Time PCR System (Applied Biosystems, United States). Gene transcript levels were determined according to the 2^–ΔΔCt^ method, using 16s RNA as a reference gene for normalizing the gene expression levels. Reactions were performed according to the manufacturer’s instructions, and three technical replicates with one negative control were performed for each sample. Values and error bars represent the mean and the s.d. ^∗∗∗^*p* < 0.001, ^∗∗^*p* < 0.01, ^*^p < 0.05 as determined by two-tailed *t*-test.

**TABLE 3:** Genes and primers used for quantitative real-time PCR.

**Gene**	**Forward primer sequence**	**Reverse primer sequence**
*fimH*	GATGCGGGCAACTCGATT	CGCCCTGTGCAGGTGAA
*flu*	CAGCGTGGAAAAATCAGGAAGT	ACGGCTTTCTGGGTGAGTGT
*cyaA*	TGCCTGGTAGGTAGCGTTGAC	GCAGCGTACGCACTTCGTT
*csgD*	CGGAATCAGCCCTCCTTACTC	GCGCCGATACGCAGCTTAT
*luxS*	GTGTTCGATCTGCGCTTCTG	GGATCCCTCTTTCTGGCATCA
*lsrR*	CGGTGGCGTCGGTTCTT	CTGCACGCCGCGTTAAG
*ackA*	GTATTTGACACCGCGTTCCA	GGCAGGGCGTAGAGGTAAGA
*aspA*	TGCAGGCGGGCTCTTC	TCCGGAACAACCGGGTTT
*lysA*	TCTCACCGCCGAAAATCTG	ACACCGGGCAGCCAAAT
*tdcB*	ATCCAAAGTAGCGGCAACGT	CGTTGAAGTTATCACCATGCAGAA
*thrA*	CTTCACCCCCGCACCAT	ATCAGGCAAGGGATCTGGAA
*thrB*	CGAGCTGGAAGGCCGTATC	AACACGGTGCCACGTTGTC
*thrC*	AAGCGACTCAGGCGACGTTA	CACACGCGGCCAGTTGT
*rhtA*	TCGTCGCCCGGTAGATTTC	GCAGGAACCACAGACCAAGAA
*16s RNA*	TCGGGAACCGTGAGACAGG	CCGCTGGCAACAAAGGATAAG

## Results and Discussion

### Characterization of Biofilm Formation in Engineered Strains

**FIGURE 2:**
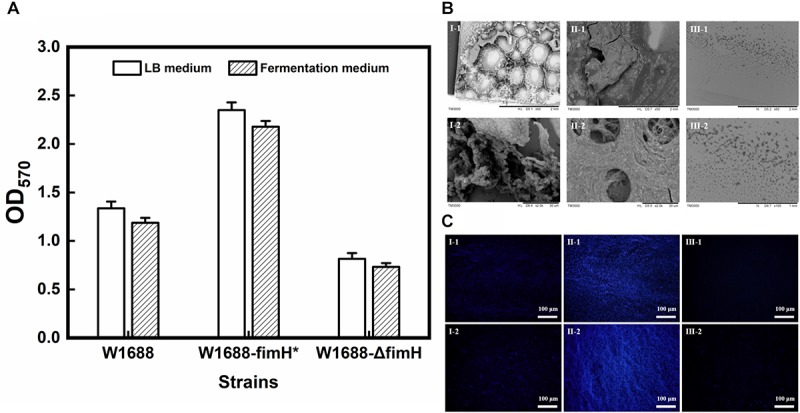
Quantitative analysis for biofilm formation in the three strains with different characterization methods. **(A)** Crystal violet staining for LB medium and fermentation medium; **(B)** Scanning electron microscope (SEM) images of biofilm formation and cell adhesion under different magnification levels; and **(C)** Fluorescence microscope images of biofilm and cell adhesion under different magnification levels. I: *E. coli* W1688, II: *E. coli* W1688-fimH^*^, and III: *E. coli* W1688-ΔfimH.

PCR and sequencing results confirmed that recombinant strains, in which *fimH* gene was overexpressed (*E. coli* W1688-fimH^*^) or knocked out (*E. coli* W1688-ΔfimH) were constructed successfully. The 96-well plates experiment showed that the biofilm formation abilities of these strains were different. The optical density from crystal violet staining (which was an indicator of biofilm amount) for *E. coli* W1688-fimH^*^ in LB medium increased greatly by 75.9% compared with that of the original strain (1.34 vs. 2.35), which could be attributed to the overexpression of *fimH* gene (Figure 2A). On the contrary, the optical density of *E. coli* W1688-ΔfimH was decreased by 38.8% due to the deletion of *fimH* gene (1.34 vs. 0.82). Similar results were also observed in fermentation medium. Furthermore, SEM and fluorescence microscope images showed that biofilm formation and cell adhesion were more obvious in *E. coli* W1688-fimH^*^ compared with the original strain (Figures 2B,C). In *E. coli* W1688-ΔfimH, biofilm formation was apparently reduced and a sparse bacterial distribution was observed. Taken together, these results indicated that overexpression of *fimH* gene facilitated cell adhesion to abiotic surfaces and contributed to the clustering effects of *E. coli* and resulted in the biofilm formation. Whereas, deletion of *fimH* gene had a negative effect on the biofilm formation. So, the *fimH* gene had a significant regulatory effect on *E. coli* biofilm formation.

### Biofilm-Based Fermentation for Enhanced L-Threonine Production

**FIGURE 3:**
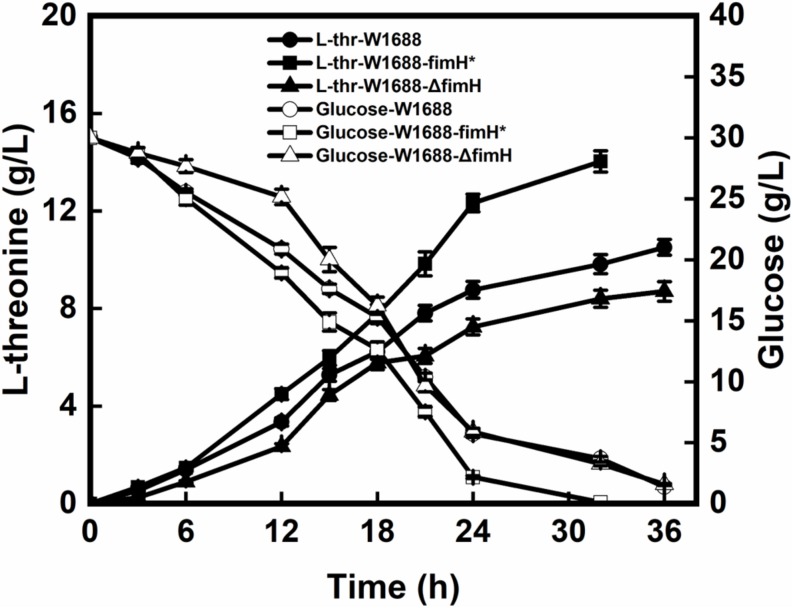
L-threonine production and glucose consumption in batch fermentation by *E. coli* W1688, *E. coli* W1688-fimH^*^, and *E. coli* W1688-ΔfimH.

The recombinant and original strains with different capabilities for the biofilm formation were investigated in batch fermentations. As seen in Figure 3, L-threonine production was increased by 42.9% in *E. coli* W1688-fimH^*^ compared with that in the original strain (14.1 g/L vs. 10.5 g/L). Besides, the fermentation period was shortened from 36 h to 32 h. In contrast, L-threonine production in *E. coli* W1688-ΔfimH showed a decrease compared with the original strain (8.7 g/L vs. 10.5 g/L) as well as a delay in glucose consumption at the initial phase of fermentation. Also, the final cell density showed a reduction of 21% compared with the *fimH* overexpression strain. Since some enzymes involved in L-threonine biosynthesis, the different level of expression in three strains might affect cell growth. All these observations suggested that L-threonine production and productivity were enhanced in strain *E. coli* W1688-fimH^*^.

**FIGURE 4:**
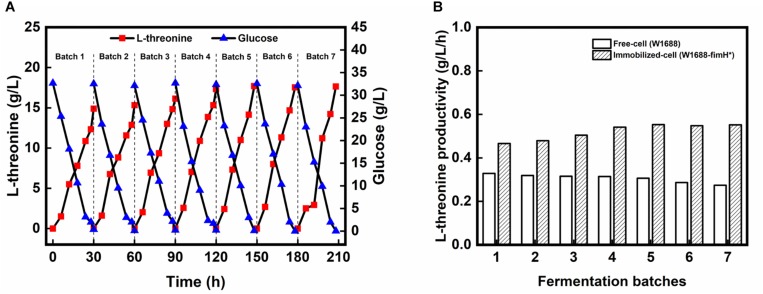
**(A)** L-threonine production by *E. coli* W1688-fimH^*^ in continuous immobilized fermentation. **(B)** Comparison of L-threonine productivities in continuous (repeated batch) immobilized fermentation with those in free-cell fermentation.

To further improve the fermentation efficiency, a biofilm-based immobilized fermentation strategy was developed. The polyurethane carrier, which could be beneficial to cell aggregation owing to its high strength and toughness was used to support the biofilm (Zhao et al., 2015). The pore size of the carrier was also important for biofilm immobilization (Yu et al., 2018). Here, immobilized fermentations by above-mentioned three strains were carried out with 10 mm × 10 mm × 10 mm polyurethane sponge pieces. In the immobilized continuous (repeated-batch) fermentation, L-threonine production in the first four batches was improved gradually in strain *E. coli* W1688-fimH^*^ (Figure 4A), while L-threonine production did not show obvious improvement in strain *E. coli* W1688 (around 10.4 g/L) and *E. coli* W1688-ΔfimH (around 9.5 g/L; Data not shown). After the 4th batch, L-threonine production was maintained at an average of 17.5 g/L during a fermentation period decreased from 30 to 28 h. L-threonine productivity was kept at about 0.63 g/L/h from fourth batch, which was much higher compared with that from free-cell fermentation by the original strain (0.63 g/L/h vs. 0.35 g/L/h) (Figure 4B). Near 1-fold improved productivity contributed to a less time in a batch fermentation and we could achieve more products in the same fermentation time. These indicated that the continuous (repeated-batch) immobilized fermentation strategy taking advantage of biofilm formation in *fimH* overexpression strain could enhance L-threonine titer and productivity.

**FIGURE 5:**
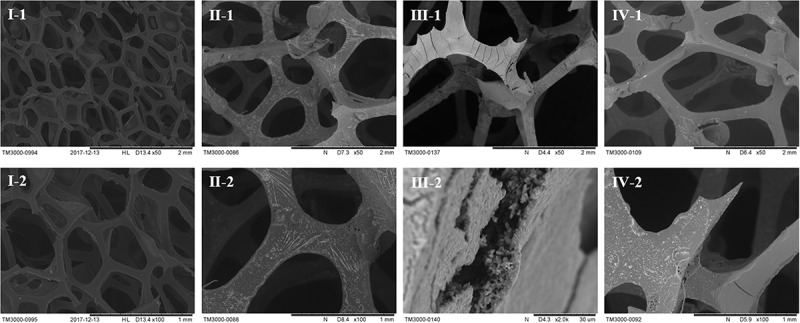
Scanning electron microscopy images of carrier in the immobilized fermentation by three different strains. I: fresh carrier, II: *E. coli* W1688, III: *E. coli* W1688-fimH^*^, IV: *E. coli* W1688-ΔfimH.

To further confirm biofilm formation by strain *E. coli* W1688-fimH^*^ under industrial fermentation conditions, scanning electron microscope experiments were performed. The images of carriers during the immobilized fermentation with different strains are shown in Figure 5. Biofilm formation could be observed obviously when using *E. coli* W1688-fimH^*^. It could be concluded that the carrier could fix bacterial cells on the surface and provided good conditions for oxygen- and mass-transfer during the cell growth process (Lan et al., 2013). Furthermore, it was shown that the carrier could provide surfaces for cell adhesion and facilitated biofilm formation during the fermentation process. Hence, seed culture was avoided before each batch of fermentation owing to the existence of cells in the biofilm. In contrast, strains *E. coli* W1688 and *E. coli* W1688-ΔfimH did not show noticeable adhesion and biofilm formation on the surface (Figures 5II,IV). As a result, the immobilized fermentation by *E. coli* W1688-fimH^*^ biofilm could be continually operated to produce L-threonine. In such a fermentation mode, cell degeneration and cell growth were supposed to be in an equilibrium, suggesting that an ideal state of balance was achieved (De Ory et al., 2004). This combination of biofilm and immobilized fermentation generates a new idea, which is also applicable to other industrial fermentation processes.

### Transcriptome Analysis for Enhanced Biofilm Formation

**FIGURE 6:**
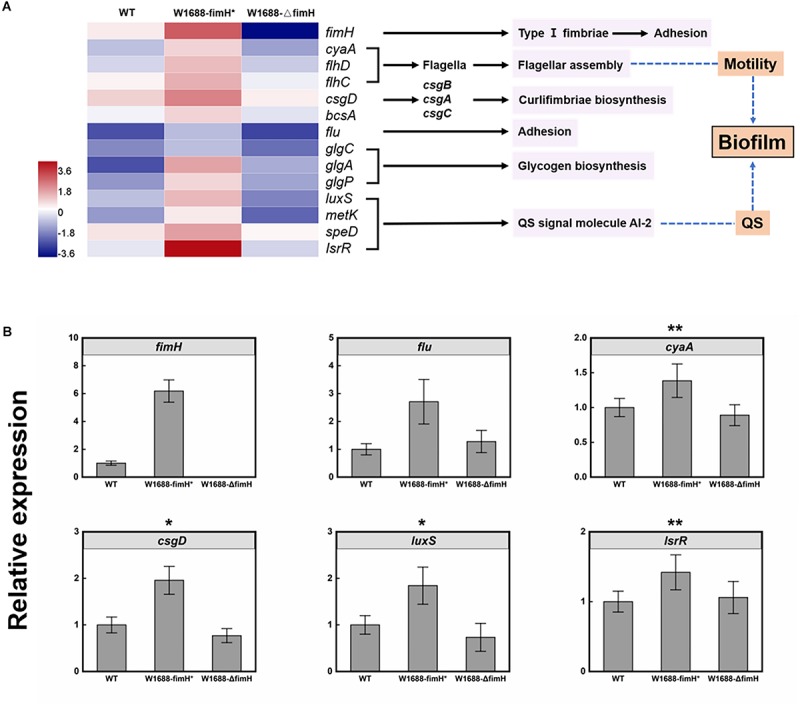
**(A)** Transcriptome analysis of genes involved in biofilm biosynthesis pathway in *E. coli* W1688, *E. coli* W1688-fimH^*^, and *E. coli* W1688-ΔfimH. Red and blue indicate up- and down-regulated genes, respectively. **(B)** qRT-PCR verification of the genes related to biofilm biosynthesis. Values and error bars represent the mean and the s.d. (*n* = 3). ^∗∗∗^*p* < 0.001, ^∗∗^*p* < 0.01, ^*^p < 0.05 as determined by two-tailed *t*-test.

To investigate the mechanism of enhanced biofilm formation, transcriptome analysis was performed for wild-type, *E. coli* W1688-fimH^*^ and *E. coli* W1688-ΔfimH. A total of over 22.7, 23.0, and 21.3 million raw reads were obtained, respectively. The expression ratios of genes involved in biofilm biosynthesis which showed significant differences among these strains were calculated in Figure 6A, where the regulated genes could be classified into six distinct clusters. The *fimH* (encoding type I fimbriae adhesin) and *flu* genes guide the secretion of adhesins responsible for cell adhesion to surfaces (Reisner et al., 2003; Schembri et al., 2003). *FlhD* and *FlhC* are transcriptional activators involved in flagellar assembly and regulon, which is related to cell motility and biofilm formation (Beloin et al., 2004). *CsgD* is in charge of curli assembly, transport and structural components biosynthesis for biofilm formation together with *csgA*, *csgB*, and *csgC* (Reisner et al., 2003). The *glgA*, *glgC*, and *glgP* control glycogen biosynthesis (Beloin et al., 2004). The *luxS*, *metK*, *speD*, and *lsrR* are involved in biosynthesis of quorum sensing (QS) signal molecule AI-2 (autoinducer-2), which can activate transcription factors to promote formation of biofilm when the cell density reaches to a threshold (Vendeville et al., 2005). Actually, gene *luxS* concerning catalyzing the reaction to AI-2 was widely spread in Gram-negative and -positive bacterium, which shows a high homologous conservation. All these genes were up-regulated in varying degrees in the *fimH*-overexpressed strain *E. coli* W1688-fimH^*^, while they were down-regulated in the *fimH*-deleted strain compared with the wild-type. Concentration of extracellular AI-2 could also be decreased rapidly by *lsr*’s ABC (ATP-binding cassette) transporter, which can transport AI-2 into the cell. The transcriptome data showed that some genes in *lsr* operon were down-regulated, which might further lead to accumulation of extracellular AI-2 and promote the expression of biofilm-related genes (Schauder et al., 2001; Barrios et al., 2006). The genes showed various degrees of upregulation in *fimH* overexpression strain, which was validated by qRT-PCR analysis (Figure 6B). Notably, the transcription level by *fimH* was 6.2-fold higher than that in *E. coli* W1688-fimH^*^ compared to the wild type, which was close to 0-fold in *fimH* deletion strain, indicating that the deletion of *fimH* was successful in *E. coli* W1688-ΔfimH. Besides, *flu* gene expression showed more than 2-fold in *E. coli* W1688-fimH^*^ by qRT-PCR analysis. *CyaA*, *csgD*, *luxS*, and *lsrR* involved in flagellar assembly and signal secretion and reception of quorum sensing were up-regulated with more than 1-fold with original strain, while these genes showed down-regulated with varying degrees in *E. coli* W1688-ΔfimH. Taken together, the overexpression of *fimH* gene triggered modulation of related genes to enhance biofilm formation in *E. coli* W1688-fimH^*^.

### Transcriptome Analysis for Overexpressing *FimH* Gene to Increase L-Threonine Production in *E. coli*

**FIGURE 7:**
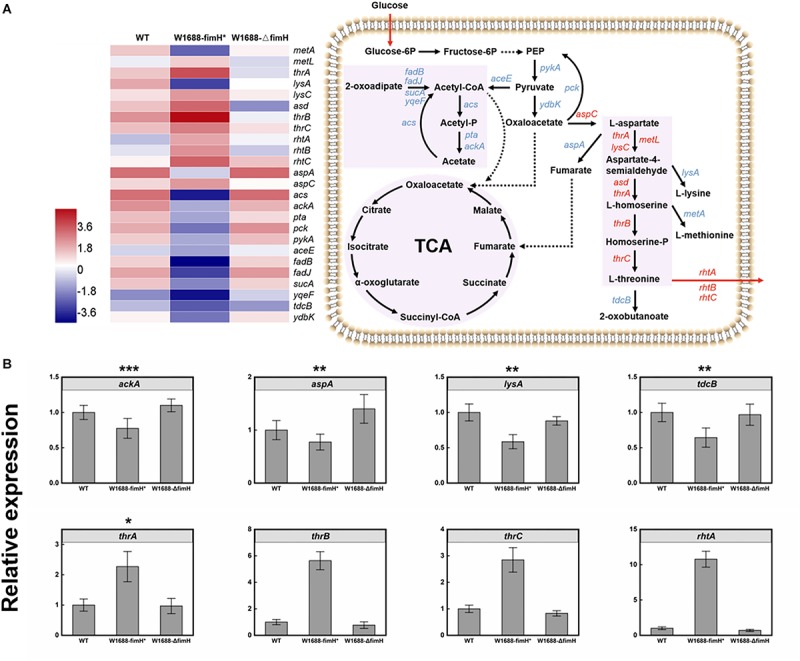
**(A)** Transcriptome analysis of L-threonine biosynthesis pathway genes in three different strains and the expression levels of these genes in *E. coli* W1688-fimH^*^. Red and blue indicate up- and down-regulated genes, respectively. **(B)** qRT-PCR verification of the genes related to L-threonine biosynthesis and transportation. Values and error bars represent the mean and the s.d. (*n* = 3). ^∗∗∗^*p* < 0.001, ^∗∗^*p* < 0.01, ^*^p < 0.05 as determined by two-tailed *t*-test.

The overexpression of *fimH* resulted in oversecretion of adhesion protein, which was beneficial for the gather of cells to biofilm production. Moreover, the immobilized fermentation system based on biofilm formation for L-threonine production was applied in increasing L-threonine production. To figure out whether the increase in L-threonine production was also associated with regulation of biosynthetic pathway genes and elaborate the molecular mechanism linking biofilm formation to L-threonine biosynthesis, further transcriptome analysis was performed. Fortunately, it was found that overexpression of *fimH* gene resulted in not only the accumulation of biofilm, but also regulation of genes in the L-threonine biosynthetic pathway (Figure 7A). The key genes such as *thrA*, *lysC*, *metL*, and *asd* that dominate the pathway from L-aspartate to L-homoserine, and *thrB* and *thrC* that dominate the pathway from L-homoserine to L-threonine (Lee et al., 2003; Livshits et al., 2003) were all up-regulated by an average of 11-fold in *E. coli* W1688-fimH^*^ compared with those in wild-type strain and *E. coli* W1688-ΔfimH. The L-threonine transporter-encoding genes *rhtA, rhtB*, and *rhtC* were also up-regulated, which would facilitate the extracellular accumulation of L-threonine (Kruse et al., 2002). These results indicated that the enhanced biofilm formation affected the enzymes expression of L-threonine pathway and facilitated the central carbon flux. On the other hand, the *tdcB* gene encoding threonine dehydratase was down-regulated, which would benefit the accumulation of target products. In addition, down-regulated genes were *lysA* and *metA*, which catalyzed the last step in L-lysine biosynthesis and generated the L-methionine, respectively. More precursor substances involved in central carbon metabolism diverted to L-threonine formation. Obviously, down-regulation of these genes could beneficially facilitate the accumulation of L-threonine. Furthermore, expression of genes in competing branch pathways such as *aspA*, mediating the pathway from L-aspartate to fumarate were all decreased apparently in *E. coli* W1688-fimH^*^. Indeed, *aspA* encoding aspartase to synthesize target chemicals in TCA cycle always brings a competitive effect on carbon flux. Therefore, the overexpression of FimH adhesin protein could facilitate the redistribution of carbon flux via down-regulation of *aspA*. The gene expression levels of *acs*, *pta*, and *ackA* (Lee et al., 2006; Nahku et al., 2010) were down-regulated notably by 3.7, 5.9, and 6.1-fold, respectively. Since acetate accumulation has a detrimental effect on biofilm formation, cell growth and production, the transcriptional level in whole module of acetate pathway was down-regulated which could be beneficial for L-threonine production. As a result, it could be conducive to keeping the pH of broth relatively stable due to decreased acetate flux decreased. This would create a favorable condition for L-threonine biosynthesis and glucose consumption (De Mey et al., 2007). The genes for branched metabolic pathway showed various degrees of downregulation, while the genes related to central carbon metabolism and L-threonine transportation were significantly upregulated in *E. coli* W1688-fimH^*^, which were quantified by qRT-PCR (Figure 7B). The relative expressions of *ackA*, *aspA*, *lysA*, and *tdcB* were decreased at least 23% in *E. coli* W1688-fimH^*^, which these genes related to biosynthesis of acetate, the degradation of L-threonine and branch by-products like fumarate, L-lysine and L-methionine. The genes involved in L-threonine direct synthesis (*thrA/B/C*) showed more than 2.3-fold expressions than that in original strain. Significantly, the relative expression of *rhtA* was 10.7-fold higher in *E. coli* W1688-fimH^*^ than that in wild-type strain. It meant that more carbon flux contributed to L-threonine biosynthesis and the target product could be transported out of cell membrane easier. Meanwhile, the four genes didn’t show any obvious differences in *E. coli* W1688-ΔfimH compared with original strain. Overall, these results showed that the overexpression of *fimH* gene also enhanced the metabolic flux toward L-threonine by up- or down-regulating related pathway genes in *E. coli* W1688-fimH^*^.

### Comparison of L-Threonine Production by *E. coli* W1688-fimH^*^ With Other Studies

Currently, production of L-threonine in *E. coli* can be enhanced by metabolic engineering such as inactivation in TCA cycle, increasing glycolysis pathway flux and facilitating L-threonine central carbon metabolism. Besides these established metabolic pathway approaches and strategies, there are still several limiting factors hampering further improvement of L-threonine productivity such as fermentation strategy of optimization. *ThrB* and *thrC* are clustered with *thrA* in the *thrABC* operon, which is mainly responsible for central carbon metabolism to L-threonine (Lee et al., 2003). Increased *rhtA*, *rhtB*, and *rhtC* expression will hence L-threonine exported from intracellular to extracellular through transmembrane protein (Yuzbashev et al., 2013). Recently, fermentation optimization has been proved in improving L-threonine production effectively such as two-stage feeding strategy and fed-batch fermentation mode (Table 4). Compared to the original strain *E. coli* W1688, the cell growth and biofilm formation of *E. coli* W1688-fimH^*^ in the medium containing 30 g/L glucose was improving. Moreover, glucose was completely consumed in 28 h after 4th batch in biofilm-based immobilized repeated-fed batch fermentation. The highest yield (0.59 g/g) of L-threonine was achieved owing to the high expression of genes in the central carbon metabolism and decrease of by-products, which was a 60% increase compared to the original strain *E. coli* W1688. Since the biofilm formation performance can be varied depending on many genes, we further performed to screen and excavate potential genes to facilitate biofilm formation and increase the L-threonine production.

**TABLE 4:** Comparison of L-threonine production in engineered *E. coli* strains.

**Strains**	**Carbon source**	**Time (h)**	**L-threonine (g/L)**	**Productivity (g/L/h)**	**Yield (g/g)**	**Fermentation mode**	**References**
*E. coli* βIM4 (pBR322-thrA^r^)	Glucose	72	13.4	0.186	0.45	Batch	Miwa et al., 1983
*E. coli* TWF006/pFW01-thrA^*^BC-*asd*	Glucose	36	15.9	0.44	0.53	Batch	Zhao et al., 2018
*E. coli* TH28C (pBRThrABCR3)	Glucose	50	82.4	1.648	0.46	Fed-batch	Lee et al., 2007
*E. coli* MT201	Glucose	28	102	3.643	0.38	Fed-batch	Lee et al., 2018
*E. coli* THPE5	Glucose	40	70.8	1.77	0.404	Fed-batch	Liu et al., 2019
*E. coli* W1688	Glucose	36	10.5	0.292	0.367	Batch	This study
*E. coli* W1688-fimH^*^	Glucose	28	17.5	0.63	0.59	Repeated batch	This study

## Conclusion

An immobilized fermentation system for L-threonine production by *E. coli* was developed by taking advantages of biofilm formation. The engineered strain overexpressing *fimH* successfully enhanced biofilm formation under industrial cultivation conditions, which could also apply to continuous (repeated-batch) immobilized fermentation. L-threonine production was increased from 10.5 to 14.1 g/L using *E. coli* W1688-fimH^*^ during batch fermentations and was further improved to 17.5 g/L during continuous (repeated-batch) fermentations, with a productivity of 0.63 g/L/h. Transcriptome profiles indicated that the biofilm formation was enhanced by regulation of biofilm-related genes. Meanwhile, L-threonine biosynthesis was also enhanced by up- or down-regulating related genes in L-threonine metabolic pathway. The engineered *E. coli* W1688-fimH^*^ would be of great value for immobilized fermentation of L-threonine. This study will also provide a reference for developing more biochemical-producing processes based on *E. coli* biofilm.

## Data Availability

The reads and the HiSeq transcriptomic reads generated for *E. coli* W1688, *E. coli* W1688-fimH^*^, and *E. coli* W1688-ΔfimH, respectively, have been submitted to the BioProject database of National Center for Biotechnology Information (NCBI) under the accession numbers SRR8335002, SRR8335001, and SRR8334999, respectively.

## Author Contributions

TC and NL conceived and designed the experiments, performed the laboratory work, analyzed and interpreted the data, and drafted the manuscript. PR constructed the plasmids and strains, participated in the fermentation experiments, performed the shooting of electron microscope, analyzed the metabolic products, and performed the statistical analysis. XX, LY, and WS performed the transcriptome analysis of different strains and revised the manuscript. BY supplied the carriers. HY and PO critically revised the manuscript. YC and DL contributed to the experimental design, data interpretation, and critically revised the manuscript. All authors read and approved the final manuscript.

## Conflict of Interest Statement

The authors declare that the research was conducted in the absence of any commercial or financial relationships that could be construed as a potential conflict of interest.
